# Non-Invasive Prenatal Testing: Current Perspectives and Future Challenges

**DOI:** 10.3390/genes12010015

**Published:** 2020-12-24

**Authors:** Luigi Carbone, Federica Cariati, Laura Sarno, Alessandro Conforti, Francesca Bagnulo, Ida Strina, Lucio Pastore, Giuseppe Maria Maruotti, Carlo Alviggi

**Affiliations:** 1 Dipartimento di Neuroscienze, Scienze Riproduttive ed Odontostomatologiche, Università di Napoli Federico II, 80131 Naples, Italy; drcarboneluigi@gmail.com (L.C.); laurettasarno@gmail.com (L.S.); confale@hotmail.it (A.C.); ida.strina@unina.it (I.S.); giuseppemaria.maruotti@unina.it (G.M.M.); alviggi@unina.it (C.A.); 2 CEINGE-Biotecnologie Avanzate s.c.a.r.l., 80145 Naples, Italy; lucio.pastore@unina.it; 3 Fertility Unit, Maternal-Child Department, AOU Policlinico Federico II, 80131 Naples, Italy; francy.bagnulo@libero.it; 4 Dipartimento di Medicina Molecolare e Biotecnologie Mediche, Università di Napoli Federico II, 80131 Naples, Italy; 5 Istituto per l’Endocrinologia e l’Oncologia Sperimentale, Consiglio Nazionale Delle Ricerche, 80131 Naples, Italy

**Keywords:** non-invasive prenatal testing, prenatal screening, fetal aneuploidies, cell-free fetal DNA, fetal fraction

## Abstract

Fetal aneuploidies are among the most common causes of miscarriages, perinatal mortality and neurodevelopmental impairment. During the last 70 years, many efforts have been made in order to improve prenatal diagnosis and prenatal screening of these conditions. Recently, the use of cell-free fetal DNA (cff-DNA) testing has been increasingly used in different countries, representing an opportunity for non-invasive prenatal screening of pregnant women. The aim of this narrative review is to describe the state of the art and the main strengths and limitations of this test for prenatal screening of fetal aneuploidies.

## 1. Introduction

The purpose of prenatal diagnosis is to reduce both the incidence and prevalence of inherited conditions, which have a strong impact on both the psychological and economic aspects of people’s lives, whether the ill ones or their parents, as well as being an economic burden for national health systems. Chromosome abnormalities have been thoroughly investigated since Down syndrome was characterized as a trisomy. Technical innovations through the decades have then made it possible to detect smaller genetic anomalies, up to many single gene disorders. The non-invasive prenatal test (NIPT) is the last innovation in the field of prenatal diagnosis aimed at helping both practitioners, in the management of pregnancy and its counselling, and future parents in developing conscious and informed choices regarding their unborn child. Indeed, the aim of this narrative review is to describe the history of prenatal investigations, the technical aspects of NIPT, with its strengths and limitations, and to discuss the future directions towards which it should progress.

## 2. Evolution of Non-Invasive Prenatal Screening for Fetal Aneuploidies

Prenatal diagnoses have made enormous progress since the discovery that fetal cells could be obtained during pregnancy and analyzed to screen for genetic disorders. In the 1960s, many studies addressed the possible role of amniotic fluid cytology for determination of fetal sex and karyotyping [1,2]. Only during the 1980s chorionic villi have been sampled to perform fetal karyotyping, shifting the prenatal diagnosis from the second to first trimester [3,4]. From that moment, the use of amniocentesis and chorionic villous sampling (CVS) for prenatal diagnoses of genetic disorders has been increasingly utilized. Historically, the main limitation of these techniques was related to their invasiveness and possible procedure-related pregnancy loss [5]. Even though it is very difficult to quantify the increase in the risk of pregnancy loss after these procedures, recent meta-analyses reported that the procedure-related risk is less than 1%, but it still exists [6,7]. Therefore, the scientific community spent the last 50 years trying to identify non-invasive screening tests to select women at increased risk of fetal aneuploidies in order to limit the use of invasive tests. In the 1960s, the main indication for an invasive procedure was advanced maternal age; however, the use of maternal age as an index by itself has a very low sensitivity (around 30%) and a very high false-positive rate (FPR) (15%) [8]. Moreover, although it is true that increasing maternal age raises the risk for trisomy 21, 13 and 18 (T21, T13 and T18), it does not represent a risk factor for other aneuploidies, like sex chromosome aneuploidies or triploidy. Subsequently, identification of biochemical markers of fetal aneuploidies [9,10,11,12,13] gave birth to two different screening tests: the triple test and the quadruple test, with a detection rate (DR) of around 60% and 65%, respectively, and an FPR of 5% [8]. The real revolution in the field of prenatal screening was represented by the introduction of nuchal translucency (NT) and the combined screening test in the 1990s [14,15]. A first trimester combined screening test for trisomies 21, 18 and 13 is performed combining maternal age, nuchal translucency (NT), fetal heart rate (FHR) and the multiples of median (MoMs) of circulating free β-hCG and PAPP-A [14,15]. Additional markers, such as the ductus venosus pulsatility index (PI), nasal bone and tricuspid regurgitation can be added, in order to ameliorate the performance of the test [16,17,18]. A recent study by Santorum et al., revising more than 108,000 combined screening tests, stated that at an FPR of 4% has a DR of 90%, 97% and 92% for T21, T18 and T13, respectively [19]. Roughly 50 years ago, it was demonstrated how, despite the presence of the blood–placental barrier, it was possible to detect fetal nucleated cells in maternal circulation [20]. In 1997, Lo et al. [21] were the first to find cell-free fetal DNA (cff-DNA) in maternal plasma and serum. They demonstrated that its concentration in maternal blood increases with gestational age and it is suitable for pregnancy tests due to the fast clearance after the end of the pregnancy [22]. Among the various applications of such a discovery in the field, the most successful one has been the introduction of prenatal screening for aneuploidy [23,24]. In 2008, Fan et al. [25] and Chiu et al. [26] demonstrated how it was possible to screen for T21 by sequencing cff-DNA in maternal plasma with a very low FPR. Cff-DNA can be analyzed with a simple blood sampling from the pregnant woman [27]. Therefore, this has been called the non-invasive prenatal test (NIPT). Since its introduction in 2011, more than 2 million NIPTs have already been performed [28]. Nowadays, alongside the evolution of genetic testing, there is a growing need for the reassurance of the health of progeny, both if coming from natural conception and in vitro fertilization techniques [29,30,31]. Genetic tests have been developed also with the aim to discover the carrier status among couples, in order to reduce the chance of incurring recessive conditions in the pre-conception period [32,33]. Another option is an analysis of the blastocyst DNA before the implantation of the embryo [29,34,35,36,37]. However, pre-conceptional tests are not designed to exclude pregnancy tests in the antenatal period, such as the NIPT [38].

## 3. Available Techniques for Analysis

**Figure 1:**
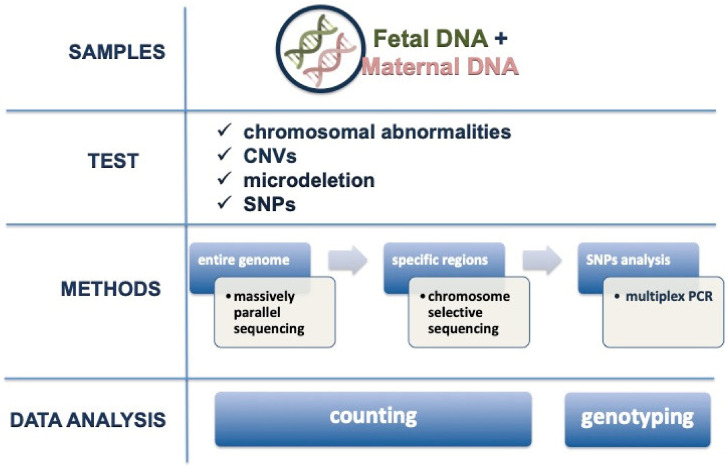
Non-invasive prenatal test (NIPT) analysis. CNVs: copy number variations, SNPs: single nucleotide polymorphisms.

The methodology used for NIPT, in order to analyze chromosomal abnormalities, copy-number variants (CNVs) and microdeletion, was developed for the entire genome, specific regions or single nucleotide polymorphisms (SNPs) analysis (Figure 1) [39]. The majority of clinical trials are conducted performing massively parallel shotgun sequencing (MPSS) and chromosome selective sequencing (CSS) [40,41]. MPSS analyzes the whole genome, sequencing millions of cf-DNA fragments, both fetal and maternal. Each fragment is assigned to the original chromosome and those from each chromosome are quantified. The amounts of fragments in a trisomic fetus will be higher than the one expected in euploid fetuses [40]. CSS restricts the sequencing to specific regions of chromosomes 21, 18, 13, X and Y. Its advantage is related to lower costs, but the limitation can be the higher (around 2%) failure rate compared to MPSS [41]. SNP analysis is based on the chance to find a difference in a single base of nucleic acid in a DNA sequence [42]. SNP analysis by a multiplex PCR allows the differentiation between maternal and fetal fragments [43]. They are used to quantify the fetal fraction (FF) in CSS or as a technique itself, showing a performance similar to MPSS and CSS, with a slightly increased failure rate (around 4%) [44]. Recently, microarray quantification has been proposed as an alternative approach; it seems to be cheaper and faster than CSS [45]. Moreover, the use of this technique removes the risk for PCR contamination and reduces the assay variability. Another technique that has been proposed is digital PCR, initially validated on trisomy 21. If compared to NGS, it seems rapid and cost-effective, but it needs adequate levels of cff-DNA [46]. The digital PCR is based on the single molecule counting strategy to detect cf-DNA. In fact, the digital PCR uses a single probe set, limiting the application in large-scale analysis and cannot detect low-grade mosaicism or chromosomal structural abnormalities such as balanced translocation. Because of these limitations, NGS is still more commonly chosen. In addition, recent data show the feasibility of the detection of micro-deletions/duplications, at a resolution comparable to microarray analysis [47]. In relation to microdeletions, there are data on Di George syndrome, Prader-Willi/Angelman syndrome, Cri-du-chat syndrome and del1p36 syndrome, but microdeletions shorter than 3 megabases are not included [48]. Furthermore, routine implementation for such indications is hampered by its requirements for significantly deeper sequencing, which is costly [49]. Whenever a patient would ask, for specific reasons, for an enlarged panel of chromosomal or genetic anomalies, it is important to clarify that neither the combined test nor the cff-DNA test are currently useful for it, and, accordingly, the patient should opt for an invasive test with microarray analysis.

## 4. Role of Fetal Fraction and Failure Rate

The FF is the amount of cff-DNA divided by the amount of total circulating cf-DNA [50]. The higher the FF in maternal blood, the more reliable is the result, because it is less difficult to differentiate maternal than fetal cf-DNA. It has been seen that, at a 4% FF, the DR is 62.1%, while above 9% it reaches 100% [51]. Based on this, many companies prefer to consider the test as failed in a case of FF lower than 4%, because of the low reliability in such situations [52]. Since the main cause of the failed results is a low FF, factors affecting it will affect also the failure rate. Other reasons for test failure, apart from a low FF, can be mistakes during blood collection or transport of the samples, and laboratory failure [53]. Cff-DNA can be detected in maternal plasma as early as 5–7 weeks [54]. It increases during gestation, from 0.1% per week between 10 and 21 weeks of gestation, to 1% per week beyond 21 weeks of gestation [55]. In order to reach a sufficient FF, NIPT should not be performed before 10 weeks [56]. Moreover, the FF seems directly related to the crown–rump length (CRL), PAPP-A and free B-hCG MoMs, and is higher in East-Asian races (China, Japan) and in smokers (mostly due to a decrease in maternal cf-DNA); on the other hand, it decreases with increased maternal age and body mass index (BMI) (due to an increase in maternal cf-DNA more than a decrease in cff-DNA), is lower in twin pregnancies, in Afro-Caribbean and South-Asian women (India, Pakistan) and in IVF pregnancies, as well as in women with a high PI of uterine arteries at the first trimester scan [57,58]. The FF is lower in twins compared to singletons and in dichorionic compared to monochorionic twins [58]; as a consequence, the failure rate is around three to four times higher in twins than singletons [59]. A higher failure rate can be related to a limitation of the test itself. A cff-DNA test is more complex in twin pregnancies, because if dizygotic, there could be the chance that only one twin carries chromosomal abnormalities [58,60]. In addition, the contribution to the FF could be different between the twins (up to 2 times difference) [61]. For example, a negative result could appear in case an affected twin contributes with an FF less than 4%, but the total FF would be more than 8%, because of the higher contribution of the healthy twin [58,62]. To avoid this risk, it has been proposed to consider the lower FF between the two fetuses, more than the total FF [62]. Moreover, the higher failure rate also can be explained by the higher incidence of IVF among twin pregnancies [58]. Interestingly, the International Society for Prenatal Diagnosis reviewed how various professional societies addressed the issue of cff-DNA screening in twins, showing that only few of them (United States, Canada, Australia-New Zealand, and Germany–Austria–Switzerland) recognized its usefulness and applicability in clinical settings, although with caution, while the majority still await further evidence [63]. In addition, it has been seen that the FF is the same or sometimes higher in T21 than in euploid cases, whereas it is usually lower in T18, T13 and triploidies [27,64,65,66]. Some studies indicate that, in the proportion of failure due to a low FF, there is a moderate rate of aneuploidies [64,65,66]. This seems valid especially for T18 and T13, where these are associated with a low PAPP-A, reflecting a smaller placental mass, and consequently reduced source of cff-DNA [27]. Due to these findings, the American College of Obstetricians and Gynecologists (ACOG) recommended a diagnostic test in such cases, rather than a repetition of the test [67,68]. Recently, ACOG released a practice bulletin on the screening for fetal chromosomal abnormalities, confirming that, when the NIPT results are not reported or uninterpretable, patients should be informed of the possibility of fetal aneuploidy, and a thorough ultrasound, genetic counselling and diagnostic test should be offered [69]. However, since the possibility to get a result after repetition of the test has been reported to be around 70% in singletons and 55% in twins, it seems to be reasonable to offer it in case of non-evident ultrasonographic fetal anomalies [59,65]. Obviously, the decision about further testing after a failed result depends on the known or supposed reasons for failure: Galeva et al. [59] showed how conception by assisted reproduction is the most important contributor to test failure, followed by ethnic origin, BMI (risk increases by 5% with each additional Kg in maternal weight), age and parity. Therefore, the possible causes must be investigated before a redraw is offered. Furthermore, when the FF is above the 95th centile, it has also been associated with complications in pregnancy, such as the delivery of small for their gestational age babies [70]. Another recent study found that, when the FF is below the 10th centile, it is associated with an increased risk of preeclampsia or preterm birth and, when it is below the 5th centile, it is associated with a low birth weight [71]. Lastly, a systematic review observed that circulating nucleic acids (including miRNA) seem to be promising predictors for late pregnancy complications, but data are still too scant to make definitive conclusions [72].

## 5. Reliability of the Test

Cff-DNA appears to originate from the cytotrophoblasts of chorionic villi. Therefore, a mosaicism confined to the placenta can be a possible cause of a false positive and this represents one of the main reasons for discordant results between NIPT and invasive tests [73,74,75,76]. Other main contributors to false-positive (FP) and false-negative (FN) results are a low FF [52], maternal chromosomes’ aberrations [77], fetal mosaicism [50,75,78], pathogenic copy-number variants (CNV) [52,78] and a vanishing twin [79,80]. The last condition can be responsible also for fetal sex of Rhesus-D (RhD) status discordance [81]. A false positive can also result from unknown maternal cancer [82]. According to a recent meta-analysis, the DR and FPR in singletons are 99.7% and 0.04% for T21, 97.9% and 0.04% for T18 and 99.0% and 0.04% for T13, respectively [83]. In twin pregnancies, the DR and FPR for T21 are comparable to singletons, while data are insufficient to affirm the same for T18 and T13 [44,58]. An updated meta-analysis of seven studies assessing the performance of a cff-DNA test in twins revealed that the DR and FPR for T21 were 98.2% and 0.05%, respectively; for T18, the pooled weighted DR and FPR were 88.9% and 0.03%, respectively; for T13, the DR was 66.7% and FPR was 0.19% [44]. Despite the high DR shown for these chromosome abnormalities, the positive predictive value (PPV) is not always as high, given that it also depends on the prevalence in the population, and it can vary according not only to age but also among laboratories. In fact, as also described by ACOG, PPV oscillates between 38–80% and 91–99% for T21, between 11–41% and 66–92% for T18, and between 5–13% and 45–71% for T13, respectively, at 20 and 40 years old [69].

Regarding sex chromosomes, the majority of reported evidence is related to monosomy X. For this aneuploidy, the DR and FPR in singletons have been reported as 95.8% and 0.14%, respectively [83]. Sex chromosome aneuploidies, included monosomy X (Turner syndrome), Klinefelter syndrome (47,XXY o 48,XXYY), triple X syndrome (47,XXX) and 47,XYY, taken together, have an overall prevalence of 1:500, and are therefore more common than major trisomies [84]. Although many cases of sex chromosome aneuploidies are characterized by a mild phenotype, without neurological or cognitive handicaps, others show a typical phenotype with physical anomalies, intellectual delay and infertility [84]. It could be important, for some couples, to have a prenatal test for such conditions, to give the opportunity for an informed choice regarding pregnancy and prognosis of the offspring, allowing also to consider termination of pregnancy (TOP). Traditional methods of screening for aneuploidies (maternal age, ultrasound markers, and biochemical factors) are not effective in recognizing sex chromosome aneuploidies, except for Turner syndrome cases, which show cystic hygroma [85]. Even though all cases with 47,XYY, 47,XXY e 47,XXX have been identified, the total number of them is too small to draw definitive conclusion on the performance of NIPT for such aneuploidies [84,85]. The decision about to screen or not for such aneuploidies, given their usually mild phenotype or the possibility to reveal unexpected maternal aneuploidies, not known until then, needs further considerations on couples’ preferences and on the clinical usefulness of such diagnostic efforts [85]. Its “collateral” effect is the reduction in efficiency of NIPT screening for the likely rise in FPR. The high incidence of mosaicisms, both maternal and/or fetal, for such aneuploidies, represents a limitation. Various studies have shown that fetal mosaicisms can overcome 50% of sex chromosomes aneuploidies [85]. Moreover, it is worth remembering that the results should be confirmed by amniocentesis (and not by CVS, due to the abovementioned mosaicisms) [84]. On the other hand, in case of maternal mosaicism, previously known or not at the moment of the test, the analysis could wrongly count fragments of the X chromosome as fetal aneuploidy, whereas the fetus is euploid [84]. Prenatal screening tests have never been directly structured and addressed to the recognition of sex chromosome aneuploidies, with a coincidental revelation in pregnancies requiring invasive test to rule out a trisomy 21. With time, the rise of prenatal screening testing DR has reduced the request for invasive procedures and, unavoidably, also the coincidental revelation of sex chromosome aneuploidies alternative to Turner syndrome [84]. Of consequence, we could argue that, for patients with a high NT in first trimester, or cystic hygroma or hydrops in the second trimester, it is better to perform an invasive test rather than the cff-DNA analysis, to include a more accurate evaluation of monosomy X syndrome [85]. Triploidies differ depending on the origin of the extra chromosomes, if paternal (diandric) or maternal (digynic) [46]. In the latter case, the placenta is very small and the fetus shows a very severe early growth restriction, with usually a normal NT and very low free β-hCG and PAPP-A (<0.1 MoM); instead, in diandric ones, the placenta appears bigger and partially molar, free β-hCG is very high (around 10 times) and NT is larger. SNP assays can identify diandric aneuploidies, and provide a suspicion of the digynic ones, given an extremely low FF [60,80,84,86].

## 6. Implementation of NIPT into Clinical Practice

There are two options to introduce NIPT into clinical practice, both for twin and singleton pregnancies: first is to perform the test to everyone at 10 weeks, followed by a first trimester ultrasound and combined test at 12 weeks [53]. So, in patients with a high-risk score, the invasive test and selective feticide (in twins) can be planned still during the first trimester. If the test fails or the results are negative, the following management can be oriented by ultrasound and combined test results. This configures the universal screening with NIPT. The alternative is the contingent test, taking into account the results of the first trimester ultrasound and combined test [53]. This approach seems to keep the main advantages of NIPT, enhancing DR and decreasing FPR, at lower costs compared to universal screening at 10 weeks [87]. The related disadvantage is the possible shift in the diagnosis, in case of failure of the NIPT, from the first to the second trimester. In such cases, immediate access to invasive testing could be proposed when the risk comes high, or the option of NIPT when the risk is intermediate [88]. The contingent test would take also the advantage of a careful ultrasound, useful for various reasons, as correct dating, exclusion of major abnormalities, being markers of aneuploidy (megacystis, holoprosencephaly, gastroschisis and omphalocele) or not (spina bifida), and early prediction of pregnancy complications, such as preterm birth or pre-eclampsia [87]. If the NIPT fails, the options are to repeat the sampling, to opt for an invasive test or no more tests (with following management based on combined test results) [65]; if the first trimester scan poses doubts of structural abnormalities, a confirmation must follow through invasive testing; oppositely, if such abnormalities are not seen at ultrasound, a second NIPT sample can be the following option [27,89]. If it fails again at the second attempt, options are, again, the invasive test or no more test. Therefore, if there are no suspicious ultrasound features in the occasion of the combined test, even after a second failure, considering the low the risk for T18 and T13 (given the very high prevalence of fetal anomalies in such trisomies), one can wait for an anomaly scan if the a priori risk for T21 is low; if, oppositely, the a priori risk for T21 is high, an invasive test is advisable (amniocentesis) [65]. There are three main limitations to the introduction of a cff-DNA test into clinical practice: the first one is the cost, which is still higher when compared to other screening tests and more or less similar to invasive tests with karyotype analysis [88]. An overall diffusion would reduce the costs but the speed and the amount of such a process of abatement are still uncertain. The second limit comes from failed results, which can cause a challenge in the management of these cases [90]. As described by Gratacos and Nicolaides in 2014 [91], when the NIPT started to be offered in clinical settings, another limit was the time to wait for the results, which was quite long, since not many laboratories were performing such an analysis, and therefore it could have led to a slide of the diagnosis from the first to the second trimester, losing the advantages obtained from the prenatal screening history in the last 30 years. However, it must be acknowledged that, now, at least for the three main chromosomal abnormalities, the waiting time is one week maximum on average.

## 7. The Challenge of a Non-Invasive Prenatal Diagnosis for Monogenic Disease

Despite the NIPT for aneuploidy still being considered a screening test, the possibility to make a diagnosis for several monogenic diseases is actually a realistic opportunity. The most common clinical applications for a non-invasive prenatal diagnosis include the fetal RhD status determination in case of RhD-negative mothers, sex determination in case of risk for sex-linked disorders and pregnancy at risk of de novo, dominant or recessive conditions. The main indications are essentially a case of known family history or abnormal sonographic findings. Techniques of exome sequencing allowed to reveal the presence of monogenic diseases after an invasive prenatal diagnosis showed a normal karyotype in fetuses with abnormalities at ultrasound [92,93]. In such situations, a comprehensive genetic counseling of the couple is demanding to define the strategy. The use of disruptive technology, such as NGS, technically allows to overcome the challenge for early diagnosis of monogenic disease (non-invasive prenatal sequencing for multiple mendelian monogenic disorders, using circulating cell-free fetal DNA). Since the sequencing of the cf-DNA includes fetal DNA, the most reliable results are based on indirect analysis [94]. The proof of concept is to use the haplotyping strategy as already used for other diagnoses, such as preimplantation genetic diagnoses [95,96,97]. The two mainly studied analytical approaches are the relative mutation dosage (RMD) and relative haplotype dosage (RHDO). The RMD requires parental genotyping and design of mutation assay. The RHDO requires information of the parental haplotype and, more specifically, the determination of the informative heterozygous SNPs linked to the mutation site. It is possible to obtain this information using an affected proband within the family as the reference or using a direct analysis of the parental haplotype by linked-read sequencing [95]. About the analysis, in RMD the comparison is made between counts at the specific mutation site; in RHDO, haplotypes are compared in the maternal plasma using multiple SNPs, thereby increasing the accuracy and reproducibility [95]. Indeed, studying the parental haplotypes as references through polymorphic regions, it is possible to make the diagnosis of a paternally inherited fetal allele that is not present in the maternal genome, as showed for cystic fibrosis, or variants in the fetus that are not present in the mother [98]. In the UK, non-invasive prenatal diagnoses is offered for several inherited disorders (autosomal dominant, autosomal recessive by paternal allele exclusion and X-linked inheritance) without confirmation through invasive tests [99]. In such a setting, the strength of the NGS is represented by the possibility to analyze several variants in one panel. In addition, the ability of NGS to analyze a single mutation site is less robust in comparison to the indirect analysis, taking into account the small amount of cff-DNA in maternal plasma [100]. Finally, the presence of a fetal transcriptome and methylome open the landscape of analysis on fetal and maternal health [101].

In case of microdeletion syndromes, it must be emphasized that the PPV of cff-DNA is still quite low, with a large dataset showing only a 13% overall PPV for the most common microdeletion syndromes, such as for Di George syndrome, Prader-Willi/Angelman syndrome, Cri-du-chat syndrome and del1p36 syndrome, depending also, as abovementioned, on the very low prevalence of these conditions [102]. In addition, there is no association with known risk factors (as age is for major trisomies), which could help in the application of such a test to the population. Indeed, Wapner et al. [103] suggested a very high negative predictive value (NPV) of NIPT for these syndromes, and, therefore, although the PPV is low, a negative result could be considered reassuring. However, since data are scanty and large-scale clinical validation studies of the general obstetric population are still needed, professional societies do not yet recommend its clinical application.

## 8. Conclusions

The main indications for an NIPT, as well as for other screening test for aneuploidies, remain an advanced maternal age, a previous child with chromosomal alterations, the presence of fetal abnormalities on ultrasound examination and a history of a genetic and/or physically inherited abnormality in a parent or family member. However, the enormous innovations in the field of genetic diagnosis led to a different approach to prenatal screenings by the future parents, with the aim of being reassured that their fetus is healthy. Because of technical issues, an NIPT remains a screening test rather than a diagnostic one. However, the growing accuracy of the methodology of analysis is promising, also for the wide diffusion of this test for the screening of monogenic diseases.

## Data Availability

Not applicable.
